# Ni/Mn metal–organic framework decorated bacterial cellulose (Ni/Mn-MOF@BC) and nickel foam (Ni/Mn-MOF@NF) as a visible-light photocatalyst and supercapacitive electrode

**DOI:** 10.1038/s41598-023-46188-8

**Published:** 2023-11-07

**Authors:** Soheila Ebrahimi-Koodehi, Farhad Esmaeili Ghodsi, Jamal Mazloom

**Affiliations:** Department of Physics, Faculty of Science, University of Guilan, Namjoo Avenue, Rasht, P.O. Box 413351914, Iran

**Keywords:** Surfaces, interfaces and thin films, Materials for energy and catalysis

## Abstract

Recently, metal–organic frameworks (MOFs) and hybrids with biomaterial are broadly investigated for a variety of applications. In this work, a novel dual-phase MOF has been grown on bacterial cellulose (BC) as a biopolymer nano-fibrous film (Ni/Mn-MOF@BC), and nickel foam (Ni/Mn-MOF@NF) using a simple reflux method to explore their potential for photocatalyst and energy storage applications. The studies showed that the prepared Mn and Ni/Mn-MOFs display different structures. Besides, the growth of MOFs on BC substantially changed the morphology of the samples by reducing their micro sized scales to nanoparticles. The nanosized MOF particles grown on BC served as a visible-light photocatalytic material. Regarding the high surface area of BC and the synergistic effect of two metal ions, Ni/Mn-MOF@BC with a lower band gap demonstrates remarkable photocatalytic degradation efficiency (ca. 84% within 3 h) against methylene blue (MB) dye under visible light, and the catalyst retained 65% of its initial pollutant removal properties after four cycles of irradiation. Besides, MOF powders deposited on nickel foam have been utilized as highly capacitive electrochemical electrodes. There, Ni/Mn-MOF@NF electrode also possesses outstanding electrochemical properties, showing a specific capacitance of 2769 Fg^−1^ at 0.5 Ag^−1^, and capacity retention of 94% after 1000 cycles at 10 Ag^−1^.

## Introduction

In this century, humankind’s evolutions make us surrounded by environmental pollution. The increasing usage of fossil fuel products as a resource of energy or disposal of wastes in nature puts the earth in danger. Therefore, finding a way to reduce the ultra-usage of these harmful components and their consequential problems pushs scientists toward finding new materials. Among these materials, metal organic frameworks (MOFs) are potentially utilized for this purpose as a new kind of porous material^1,2^. MOFs are a chain of metal ions and organic ligands connected with coordination bonds. The possibility to design diverse structures along with their high thermal and mechanical stability, and the organic functionality of MOFs provides unique properties for a variety of applications such as catalysis, gas storage, gas adsorption and separation^3–9^. Meanwhile, the ability to engineer the properties of these materials by building defects in their structure opens a new frontier for scientists to use this potential for a wide range of applications. The defects can be introduced by new linkers or guest metals in a MOF fine structure^10–15^. These modulations mainly affect the band gap energy of the MOFs, which can remarkably alter their charge transfer and catalytic properties.

Moreover, the attempts to minimize the utilization of noble metals and fossil fuel-derived carbon materials lead the world towards plant-based biopolymers. Cellulose is the most well-known natural biopolymer on the earth obtained from wood, hemp, algae, bacteria, etc., which consequently has become a key source of bio-hybrid materials for several applications, especially in water treatments and renewable energies^16,17^. Among different types of celluloses, bacterial cellulose (BC) has high purity, hydrophilicity, biocompatibility, mechanical properties, and ultrafine network structures^18^. BC can be produced by various bacteria such as *Gluconacetobacter xylinus* or *Acetobacter* xylinum. However, the lack of conductivity which is crucial for energy-related devices restricts the widespread usage of BC in such applications. Therefore, various chemical or physical modification methods have been done to overcome the weakness of this valuable biopolymer. One of these approaches is incorporating these materials with other components like conductive metals and noble metal oxides^19–22^.

The combination of biopolymers and MOFs is a novel way to derive the potential benefits of both of these materials. The fine 3D structure of cellulose offers excellent mechanical support and assistance for dispersing catalytic particles by providing a template surface to nucleate precipitation. Equally, based on the nature of MOFs materials, they offer high surface area, tunable band gap, and a simple recovery and reutilization process which contribute them to being known as sensitive visible light photocatalysts. Regarding these advantages, the photocatalytic properties of Mn based MOFs and manganese oxides decorated BC have been separately investigated. Siddiqui and his colleagues^23^ have prepared *Nigella sativa* seed-based nanocomposite (MnO_2_/BC) for the removal of organic pollutants and catalytic purposes. The experiment shows 85% of MB degraded in 2 h under sunlight irradiation. In another study, Fang et al.^24^ investigated the photocatalyst behavior of Mn-MOF under UV light. They have reported that 90.8% of MB was efficiently degraded within 70 min. Here for the first time, we have tried to combine these two elements (Mn-MOF and BC) for wastewater treatment under visible light irradiation.

Moreover, MOF usage in energy storage applications is broadly investigated in recent years. In this case, it is proven that the coexistence of two or three metals as a dopant or composition substantially improved the electrochemical properties of MOFs^25,26^. For this purpose, Han et al.^27^ have used a bimetallic metal organic framework covered on multi-walled carbon nanotubes (Mn/Ni-MOF@MWCNTs) for high-performance supercapacitor preparation. They achieved a high specific capacitance of 793.6 Fg^−1^ at 1 Ag^−1^ in 1 M LiOH aqueous solution, and good cycle stability with capacitance retentions above 74.9% after 1000 continuous charge–discharge cycles. Another report by Jiao et al.^11^ revealed an enhancement in the conductivity of the Ni-MOF by inserting Co and Zn ions for electrochemical applications. The mixed metallic electrode showed an improvement in surface area and pore size which led to an outstanding energy and power density of 49.5 Whkg^−1^ and 1450 Wkg^−1^, respectively.

To the best of our knowledge, for the first time, we successfully synthesized Mn and Ni/Mn-based MOFs and MOFs-decorated BC via the facile reflux method. The novel two metallic material showed a combination of two phases in their structures, and distinct morphologies. Among the prepared samples, MOFs@BC utilized as photocatalyst under visible light irradiation in which Ni/Mn-MOF@BC performed by far a better organic dye removal than Mn-MOF@BC. Moreover, the high efficiency of Mn and Ni/Mn-MOF@NF in energy storage applications was confirmed through electrochemical analysis by showing high specific capacity, cyclability, stability, and very low electrochemical charge transfer resistance.

## Experimental

### Materials

Manganese (II) chloride (MnCl_2_), nickel (II) chloride hexahydrate (NiCl_2_·6H_2_O) and terephthalic acid (H_2_BDC, C_8_H_6_O_4_) were purchased from Merck company, N,N-dimethylformamide (DMF, C_3_H_7_NO) was provided from SAMCHUN and bacterial cellulose nanofiber sheet (≥ 99%) was produced in Nano Novin Polymer Company.

### MOF preparation

The samples were synthesized via the facile reflux method. Firstly, 50 mmol of MnCl_2_ and NiCl_2_·6H_2_O (0 and 50 mol%), and 50 mmol of H_2_BDC were dissolved in 60 ml of DMF and 40 ml of deionized water (DI). The mixture was refluxed at 110 °C for 8 h. Then, after reaching room temperature, the white and light green particles were collected by filtration. The particles were washed with DMF and DI several times to remove extra ions and ligands, and dried at 70 °C for 7 h. The fabricated species were labeled by Mn and Ni/Mn-MOF for 0 and 50 mol% Ni incorporations.

### MOFs@BC preparation

MOF @ BC was prepared by one-step synthesis. In a typical procedure, bacterial cellulose thin film was cut into small pieces and washed with DI several times. Then the clean BCs were added to the solution containing of 50 mmol of MnCl_2_ and NiCl_2_·6H_2_O (0 and 50 mol%) in 60 ml of DMF and 40 ml of DI. This mixture was stirred for about half an hour at room temperature, and then the ligand (50 mmol of H_2_BDC) was added into the container. Then, the reflux procedure was done (110 °C for 8 h). Finally, the cellulose-covered MOF slices were taken out, washed with the solvents several times, and dried at 70 °C overnight.

### MOFs@NF preparation

For the synthesis of Mn and Ni/Mn@NF, the nickel foams (NF) were ultrasonically cleaned in HCl and ethanol solutions for 30 min, consecutively, and after that were situated in the solution container during the reflux process. Finally, the samples were dried at 70 °C for 7 h.

### Characterization

X-ray powder diffraction measurement (PXRD) was directly carried out by EQ 0434520 31 15: Bruker D8 Advance in a reflection mode with Cu (Kα_1_,_2_) radiation in 2*θ* range of 3 to 80° with 0.019 steps. An Alpha-Bruker spectrophotometer was exploited to record Fourier transform infrared spectroscopy (FTIR). The morphology and elemental analysis of the nanoparticles were investigated using a MIRA3 TESCAN-XMU field emission scanning electron microscope (FESEM) instrument with an accelerating voltage of 15 kV. Before FESEM measurement, the samples were coated with gold through sputtering technique. Transmission electron microscopy (TEM) images were collected by JEOL JEM 1400 with an acceleration voltage from 40 to 120 kV. Optical properties of the material were carried out using A Scinco s-4100 diffuse reflectance spectrophotometer (DRS). Thermogravimetric analysis (TGA) measurements were done by SDT Q600 V20.9 Build 20 from 30 to 900 °C under N_2_ flow with the rate of 100 mL/min.

The photocatalyst behavior of MOF@BC samples was measured by monitoring the degradation of MB under visible light. The source of the irradiation was a 7502-W lamp. To do the test, firstly, 0.2 g of MOF@BC samples were immersed in 50 mL of 10 ppm MB solution and placed in a dark room to reach a dynamic adsorption–desorption equilibrium. Then, the containers were placed under the light source at a distance of 10 cm, and during the irradiation, every 15 min, 2.5 mL of MB solution was withdrawn for recording its absorbance spectra by Varian-Cary 100 UV–Vis spectrophotometer.

The electrochemical studies of the fabricated samples were performed by a three-electrode system in a Zahner-Zennium Potentiostat–Galvanostat electrochemical analyzer using Ag/AgCl as a reference electrode and platinum wire as a counter electrode. MOFs@NF was used as working electrode. To obtain the active material mass participating in the electrochemical reaction, the mass of the Ni foam was measured before and after putting it in the reaction container by a high-precision analytical balance with 0.1 mg sensitivity (Kern ABT100-5NM). The electrochemical measurements were carried out in a 3 M KOH aqueous solution as an electrolyte. The cyclic voltammetry (CV) curves were recorded in the potential range of 0–0.6 V in different sweep rates. Galvanostatic charge–discharge (GCD) analysis was also conducted at different applied current densities in the potential window of 0–0.4 V. In addition, the electrochemical impedance spectroscopy data (in an AC voltage of 0.01 V and a frequency range of 0.01 Hz to 100 kHz) were investigated by fitting the Nyquist plot using Zview software.

## Results and discussion

**Figure 1:**
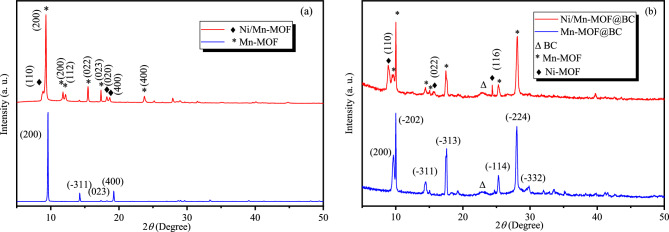
XRD pattern of (**a**) MOFs and (**b**) MOFs@BC.

The structure of the prepared MOFs and MOFs@BC was studied by PXRD measurement and shown in Fig. 1a and b, respectively. The profile matching of the patterns with CCDC numbers was conducted and is provided in the supplementary file (Fig. S1). As it is obvious, the structure of well-defined Mn-based MOF was achieved in this work, which properly matched with CCDC no. 663973 with monoclinic structure and C 2/c space group. The dominant orientations of the crystals were located at 2*θ* of 9.6, 14.3, and 19.3° related to the (200), (− 311), and (400) planes, respectively. The patterns also showed that the addition of Ni ions remarkably changed the orientation of the preferred peaks in Mn-MOF, which means that Ni suppresses the crystal growth of Mn-MOF. The trace of Ni-MOF phase (CCDC no. 1911012)^28,29^ with some peaks at about 8.9, 18.2 and 18.6° emerged in the structure of Mn-MOF. These peaks corresponded to (110), (020) and (400) plans of monoclinic structure, respectively.

Interestingly, PXRD patterns of MOFs grown on BC nanofibers have shown different orientations compared to their powder forms (Fig. 1b, S1). Therefore, in Mn-MOF@BC, some additional plans emerged at 10.0, 14.5, 17.6, 25.3 and 28.0° which corresponded to (− 202), (− 311), (− 313), (− 114) and (− 224) plans in the CCDC no. 663973, respectively. The same happened with Ni/Mn-MOF@BC in which the dominant phase was related to Mn-MOF, while three extra peaks at about 8.9, 15.6 and 24.4° represented (110), (002) and (116) plans of Ni-MOF phase (CCDC no. 1911012), respectively. Furthermore, the crystallinity of the bacterial cellulose was clarified with a shadow peak at 22.8° related to the (110) plan^20^.

FTIR spectra exhibited the chemical bonding conditions of the MOFs (Fig. S2), showing all the functional groups of BDC-MOF obtained in this experiment. It was reported that generally the absorbed bands at around 600–1300 cm^−1^ were assigned to the out-of-plane vibration of the BDC linker^30^. The graph showed two identified bands at 751 and 817 cm^−1^ in all the samples, which represented good coordination of metal ions with BDC^2−^ ligands ^31,32^. The stretching vibration bands of C–O and C–N appeared at around 1020–1150 cm^−1^^33^. The asymmetric and symmetric stretching modes of the carboxylate (–COO–) group were observed at about 1544 and 1383 cm^−1^, respectively^32^. The stretching vibration bands between 3250 and 3500 cm^−1^ indicated the presence of H_2_O molecules^34^.

Thermogravimetric analysis (TGA) was utilized to determine the thermal stability of the samples (Fig. S3). The measurements were conducted from 30 to 900 ℃ under N_2_ flow with the rate of 10 °C/min and 100 mL/min, respectively, and it shows that MOFs encountered weight losses at three different points representing different stages of transformations. A low weight loss (3.8%) was observed below 160 °C in Ni/Mn-MOFs, which corresponds to the evaporation of solvents, and absorbed water from the atmosphere. The second drop in the weight (31.8%) was also observed from 160 to over 310 °C, which is related to the incineration of excess organic H_2_BDC linker present as a host and the coordination water^35,36^, showing high thermal stability of the MOFs. A dramatic loss in the weight of the MOF (32.5%) occurred between 310 to 520 °C where the organic framework of the materials collapses^35–37^. These weight loss percentages were about 13.1, 5.2 and 41.9% for Mn-MOF, respectively.

**Figure 2:**
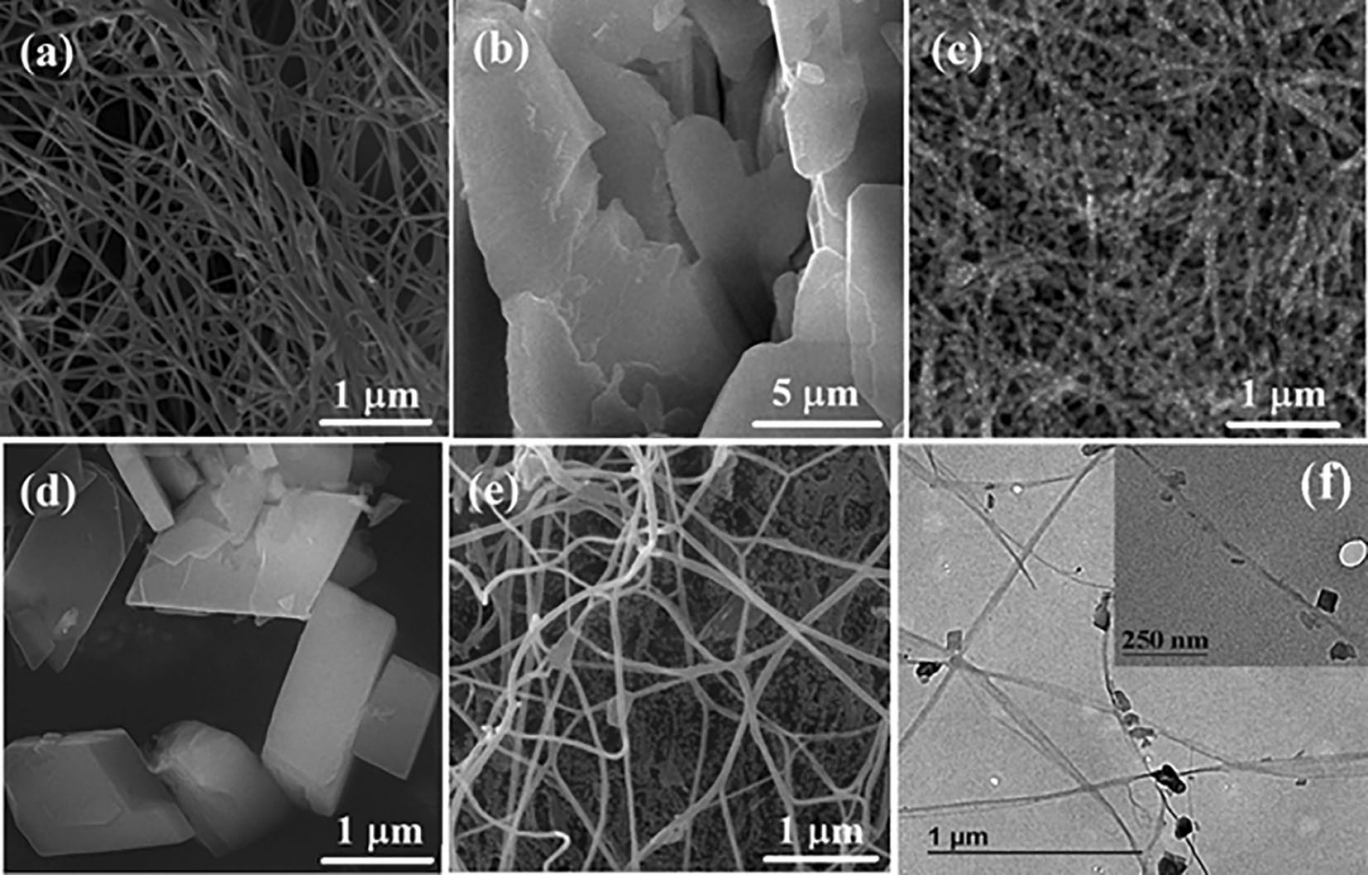
FESEM images of (**a**) plain BC, (**b**) Mn-MOF, (**c**) Mn-MOF@BC, (**d**) Ni/Mn-MOF, (**e**) Ni/Mn-MOF@BC and (**f**) TEM image of Ni/Mn-MOF@BC.

The morphology of the plain BC, fabricated MOFs and MOFs@BC were displayed in Fig. 2. As evident from FESEM pictures, BC was formed of finely structured nanofibers (Fig. 2a). Figure 2b showed Mn-MOF particles consisted of thin interconnected 2D sheets, while Ni/Mn-MOF species (Fig. 2d) had completely different features with micro-sized octagonal geometric shapes. However, when MOF particles were deposited onto BC nanofibers, both the size and morphology of the MOFs underwent significant alterations. There, nanoscale MOF particles uniformly covered BC nanofibers in both Mn-MOF@BC (Fig. 2c) and Ni/Mn-MOF@BC (Fig. 2e). As FESEM images revealed an agglomeration in MOF particles occurred in the samples, which can be occurred during the reflux method due to several reasons, like high concentration of reactants, slow nucleation and growth, solvent evaporation, surface interactions, and some instrumentation factors such as charging effects or beam-induced heating that can also contribute to particle agglomeration. TEM image of Ni/Mn-MOF covered BC was shown in Fig. 2 f. It also indicates that MOFs were successfully grown on BC nanofibers. Figure S4 exhibited EDS elemental mapping images of Ni/Mn-MOF@BC. The images showed the elements (O, C, N, Mn and Ni) uniformly distributed over the sample.

UV–Vis diffusion reflectance spectra were utilized to evaluate the optical properties of MOFs@BC (Fig. S5a). The optical band gap energy of the samples was evaluated through Kubelka–Munk (K–M) method^38^, in which K-M function is ${\text{F(}}R) = {{(1 - R)^{2} } \mathord{\left/ {\vphantom {{(1 - R)^{2} } {2R}}} \right. \kern-0pt} {2R}}$, where *R* is the diffusion reflectance. Generally, the absorbance coefficient (*α*) has a direct correlation with F(R) $\left( {\alpha = {{F(R)} \mathord{\left/ {\vphantom {{F(R)} t}} \right. \kern-0pt} t}} \right)$, where *t* is the thickness of the sample^39^. The optical band gap energy (*E*_g_) of the sample was calculated by plotting (*α*h*v*)^2^ versus incident photon energy (h*v*). *E*_g_ is the obtained value of the extrapolated linear part of the curve to (*α*h*v*)^2^ = 0 (Fig. S5b). The calculated *E*_g_ for Mn-MOF@BC and Ni/Mn-MOF@BC were 2.75 and 2.33 eV, respectively. This band gap shrinking of Ni/Mn-MOF may refer to the formation of some defects (e.g., vacancies and interstitial atoms) or structural disorientation in the structure of MOF^40^.

**Figure 3:**
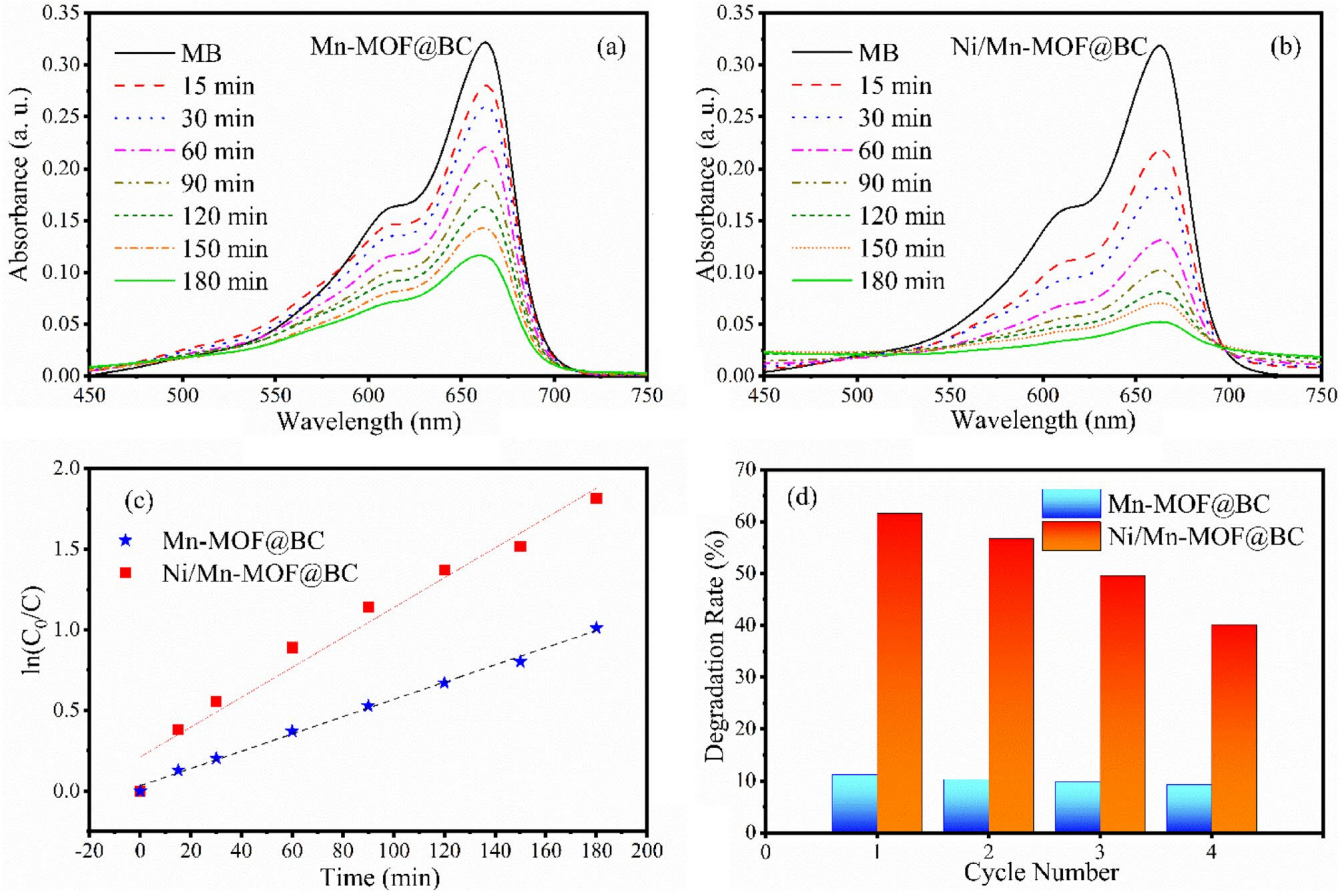
Photodegradation of MB under visible light with the presence of (**a**) Mn-MOF@BC, (**b**) Ni/Mn-MOF@BC, (**c**) kinetics of the reactions, and (**d**) photocatalyst reusability test of the MOFs@BC.

The photocatalytic performance of MOFs@BC was carried out by monitoring the degradation of MB under visible light. The amount of the remaining organic dye in water under visible light radiation was determined through recorded absorbance spectra (Fig. 3a and b). The remarkable reduction of absorbance peak of MB at 663 nm over time indicated that MOFs@BC active catalysts successfully removed the pollutant. The comparison of the graphs exhibited the removal rate of MB in Ni/Mn-MOF was by far faster than the other sample. The photodegradation rate was calculated via the following equation^41^:

1 $${\text{Degradation rate }}\% = \frac{{C_{0} - C}}{{C_{0} }} \times 100$$

where *C*_0_ is the initial and *C* is the remaining concentration of MB in the aqueous solution. It revealed that in Ni/Mn-MOF over 50% of the organic dye degraded just at the first 30 min of irradiation, while it took 120 min for Mn-MOF@BC to eliminate this amount of pollutant. The photodegradation rate of MB by Mn and Ni/Mn-MOF decorated BC after 3 h was calculated to be over 64 and 84%, respectively. Also, the kinetic of the reactions was calculated with the following relation:

2 $$\ln \left(\frac{{C_{0} }}{C}\right) = Kt$$

where *K* is the first-order rate constant (h^−1^). The results of the calculation are represented as a graph based on ln(*C*_0_/*C*) versus time in Fig. 3c. The obtained rate constants of two Mn and Ni/Mn-MOF covered BC were 0.32 and 0.56 h^−1^, respectively, which indicated the faster response activity of Ni/Mn-MOF. The photoactivity of the prepared samples could be attributed to two dominant factors. On one hand, cellulosic materials are highly hydrophilic because of electron-rich hydroxyl groups on their structures. This asset facilitates the interaction of catalyst components (attached particles) through hydrogen and electrostatic interaction, contributing to its function against pollutants by providing a higher surface area^18^. The other factor is the band gap modulation induced by Ni^2+^, and the reduction of it from 2.75 to 2.33 eV, which facilitates the electron transition during photon absorption.

Based on the photocatalytic mechanism in semiconductors, visible light irradiation excites electrons (e^−^) of the valence band (VB) and transfers them to the conduction band (CB), generating the same number of holes (h^+^‏) in VB. Then the combination of e^−^ with adsorbed O_2_ on the surface of the catalyst would lead to the formation of oxygen radicals (**·**O_2_^−^) and consequently transform to hydroxyl radicals (**·**OH). Meanwhile, **∙**OH also could be produced by the interaction of the hydroxyl groups (OH^−^) of the catalyst surfaces with h^+^‏. Finally, **·**OH helps the decompositions of the organic dye. When a photon with equal or greater energy than optical band gap of 2.33 eV irradiates the catalyst material in the polluted water, electrons would transition from the highest occupied molecular orbital (HOMO) to the lowest unoccupied molecular orbital (LUMO) of MOFs, which leads to leaving h^+^ in HOMO. Consequently, electron can transfer to O_2_ and form **·**O_2_^−^, and h^+^ in HOMO will generate **·**OH by oxidizing of hydroxyl group/water, leading to the degradation of MB^42,43^. Here, oxygen and nitrogen 2p bonding orbitals interpret HOMO and empty transition metal (Ni and Mn) orbitals act as LUMO^44^. The presence of hydroxyl groups and the narrower optical band gap contribute to the water purification of Ni/Mn-MOF@BC.

The reusability of the MOFs@BC for the purification of the polluted water was examined over 4 cycles of irradiation. The stability test was performed by irradiating the samples and MB mixture under visible light for 30 min, and after each cycle, the samples were washed with deionized water. The results showed that the degradation retention of MB remains at about 83 and 65% for Mn-MOF@BC and Ni/Mn-MOF@BC after four cycles of irradiation, respectively, (Fig. 3d). Although, the degradation stability of Ni/Mn-MOF is lower than Mn-MOF@BC, it still degrades a much higher amount of organic dye.

The higher responses of the Ni/Mn-MOF@BC in photocatalyst applications also can be explained by the higher surface accumulation of Reactive Oxygen Species (ROS). Generally, ROS (·O_2_^−^ and ·OH) is related to several parameters, such as the sample’s electronic structure, like band gap and surface defects, and influenced by grain size, specific surface area and porosity^45^. The enhanced ROSs resulting from a lower band gap and smaller grain size, therefore, higher surface area contributed to the better functionality of Ni/Mn-MOF@BC. Also, it is known that by existing different metals in a framework of MOFs, the synergetic effect leads to a dramatic band gap change which can substantially contribute electron/hole transfer to oxygen that leads to a high reaction rate^46^. Here, the combination of nickel and manganese ions in Ni/Mn-MOF creates a synergistic effect, providing more efficient electron transfer and promoting the generation of ROSs, which are crucial for dye degradation. According to DRS results, the narrower band gap of Ni/Mn-MOF can broaden the absorption spectrum of the material. This means that Ni/Mn-MOF can absorb a wider range of light wavelengths, including visible light, which is abundant in solar radiation. In contrast, Mn-MOF may have a narrower absorption range, limiting its effectiveness as a photocatalyst. The presence of both nickel and manganese ions in Ni/Mn-MOF can also facilitate the separation of photogenerated charges (electrons and holes), which is essential for efficient photocatalysis, as it prevents charge recombination and allows for longer-lived charge carriers, increasing the chances of dye molecule degradation.

**Figure 4:**
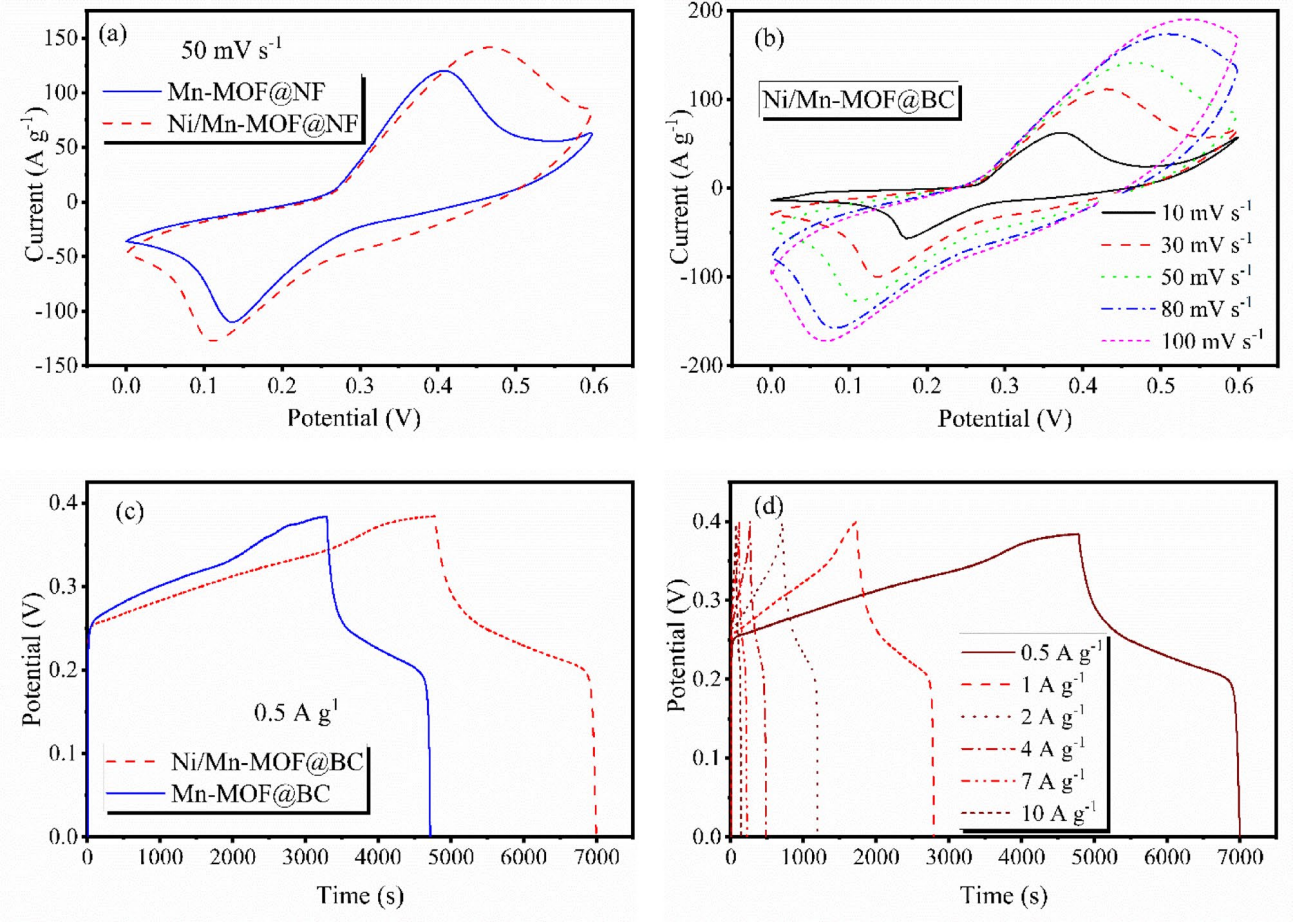
CV plots of (**a**) MOFs@NF electrodes at the scan rate of 50 mVs^−1^, (**b**) Ni/Mn-MOF@NF at different scan rates, GCD curves of (**c**) MOFs@NF at 1 Ag^−1^ and (**d**) Ni/Mn-MOF@NF at different current densities.

The electrochemical properties of the samples were examined via a three-electrode system in a 3 M KOH electrolyte. Figure 4a demonstrates the cyclic voltammetry (CV) curves of Mn- and Ni/Mn-MOF@NF at the scan rate of 50 mV s^−1^. Based on the results, all the electrodes exhibit two distinct redox peaks in the 0–0.6 V potential window, indicating the pseudo-capacitance characteristic of these electrodes. The redox peaks are attributed to changes in the oxidation states (Mn^2+^/Mn^3+^ and Ni^2+^/Ni^3+^) that take place during the electrochemical reactions, as described below^6,27^:

3 $${\text{Mn}}\left( {{\text{II}}} \right)/{\text{Ni}}\left( {{\text{II}}} \right){\text{s}} + {\text{OH}}^{ - } \leftrightarrow \left[ {{\text{Mn}}\left( {{\text{II}}} \right)/{\text{Ni}}\left( {{\text{II}}} \right)} \right]\left( {{\text{OH}}} \right){\text{ad}} + {\text{e}}^{ - }$$

4 $$\left[ {{\text{Mn}}\left( {{\text{II}}} \right)/{\text{Ni}}\left( {{\text{II}}} \right)} \right]\left( {{\text{OH}}} \right){\text{ad}} \leftrightarrow \left[ {{\text{Mn}}\left( {{\text{III}}} \right)/{\text{Ni}}\left( {{\text{III}}} \right)} \right]\left( {{\text{OH}}} \right){\text{ad}} + {\text{e}}^{ - }$$

Moreover, Ni/Mn-MOF@NF depicts a higher integral cyclic area, demonstrating its electrochemical superiority over Mn-MOF@NF. Besides, the CV curves of Ni/Mn-MOF electrode at different scan rates are performed in Fig. 4b. The graph exhibits that at higher probing rates the Faradic redox peaks are still distinguishable without remarkable change in the shape of the CV curves, which refers to the rate capability of MOFs@NF. A small shift in the redox peak positions is related to the polarization effect^47^.

Figure 4c and d display the galvanostatic charge–discharge (GCD) measurements of the MOF electrodes at 1 Ag^−1^ and different current densities, respectively. The graphs confirm that Ni/Mn-MOF by having a longer discharge time, is more capacitive than the monometallic MOF. The specific capacitance (*C*) of the electrodes was calculated via the following equation^48^:

5 $$C = \frac{I \times t}{{m \times \Delta V}}$$

where ∆*V* is the potential drop (V), *I* is the current (A) and *t* is the time of the discharge processes, and *m* is the mass of active materials (g) of the electrodes. The extracted data (Table 1) revealed that Ni^2+^ remarkably enhanced the specific capacitance of Mn-MOF@NF from 1795 to 2769 Ag^−1^, which is much higher than the reported specific capacitance of Ni/Mn-MOF prepared by other methods (Table 1S)^27,47,49^. Nickel ions can undergo reversible redox reactions easier than manganese ions. This means that Ni/Mn-MOF may exhibit better stability and efficiency in redox processes, such as oxidation and reduction reactions, which are crucial for many electrochemical applications. The superior electrochemical performance of Ni/Mn- MOF@NF also might be due to the increase of ionic defects such as vacancies and interstitial ions, leading to higher conductivity of the material^40,50^.

**Table 1:** Specific capacitance (C), Retention rate, series resistance (R_s_) and charge transfer resistance (R_ct_) of MOFs@NF electrodes.

MOF@NF	C (F g^−1^)	Retention rate (%)	R_s_ (Ω)	R_ct_ (Ω)
Mn-MOF@NF	1795	92	1.09	2.94
Ni/Mn-MOF@NF	2769	94	0.65	2.85

**Figure 5:**
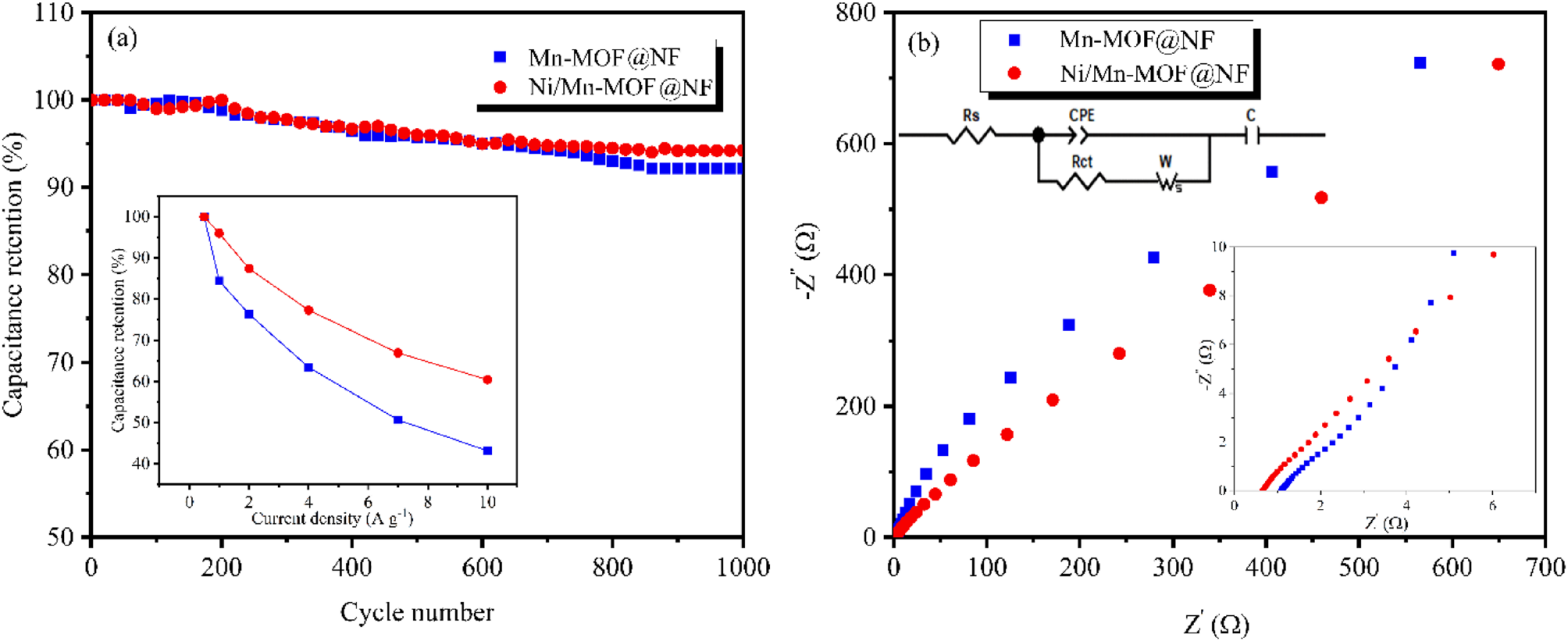
(**a**) Cyclic stability of electrodes (inset: capacitance retention at different current densities), and (**b**) EIS plots of MOFs@NF electrodes.

The specific capacitance of Ni/Mn-MOF@NF also has been calculated to be 2769, 2657, 2420, 2140, 1855 and 1675 Fg^−1^ at 0.5, 1, 2, 4, 7 and 10 Ag^−1^, respectively, which shows the excellent rate capability of the electrode by over 60% (inset of Fig. 5a). The cyclic stability of MOFs@NF electrodes at 20 Ag^−1^ over 1000 charge–discharge cycles is exhibited in Fig. 5a, and it reveals Mn and Ni/Mn-MOF@NF retain 92 and 94% of their initial specific capacitance after 1000 cycles.

Electrochemical impedance spectra were recorded to estimate the charge transfer resistance of the electrodes (Fig. 5b). The Nyquist plots show a semicircle in the high-frequency region interpreting charge transfer resistance (*R*_ct_) between the electrode and electrolyte interface, a straight line related to the Warburg diffusion resistance (*W*_s_) of the ions in the electrodes at the low-frequency parts, and the initial part of the graphs at the high-frequency region which refers to series resistance (*R*_s_) of the electrochemical setup^51^. The obtained data revealed that Ni/Mn-MOF@NF has lower *R*_s_ and *R*_ct_ by the values of 0.65 and 2.85 Ω compared with Mn-MOF@NF with 1.09 and 2.94 Ω resistances, respectively. It shows the incorporation of nickel into the framework can improve the overall conductivity of Ni/Mn-MOF, leading to better charge transfer kinetics during electrochemical reactions. In the concept of MOFs, the existence of extra inorganic nodes as bi or mixed metallic MOFs can substantially enhance the electrical conductivity of the materials, which is beneficial in supercapacitor applications. There, metal ion exchange increases the number of free carriers and interlayer distance, which leads to an enhancement in the electrical conductivity and surface area, consequently. This synergetic effect results in their electrochemical efficiency^46^.

## Conclusion

In summary, the multi-functional properties of Mn- and Ni/Mn metal organic frameworks coated on biodegradable bacterial cellulose and Ni foam substrates were investigated. The structural results confirmed the formation of two well-defined phases of MOF in Ni/Mn-MOF. The Ni^2+^ addition reduced the optical band gap of Mn-MOF@BC from 2.75 to 2.33 eV and consequently enhanced its efficiency for visible-light photodegradation of MB organic dye from 65 to 84% within 3 h. Besides, MOFs@NF showed extremely low charge transfer resistance of 2.94 and 2.85 Ω in both Mn and Ni/Mn-MOF electrodes. Ni modulation also substantially improves the electrochemical properties of Mn-MOF@NF electrode by increasing its specific capacitance from 1795 to 2769 Fg^−1^. Moreover, the Mn and Ni/Mn-MOF electrodes retained 92 and 94% of their initial specific capacitance after 1000 cycles, respectively. Overall, the results showed the Ni/Mn-MOF electrode with safe and suitable electrochemical performance is promising for practical application in energy storage devices and can play an important role in renewable energy, potentially reducing pollution and decreasing the consumption of hydrocarbon fuels.

### Supplementary Information

Supplementary Information.

## Data Availability

The datasets used and/or analysed during the current study available from the corresponding author on reasonable request.
