# Supplementary material for: Ni/Mn metal–organic framework decorated bacterial cellulose (Ni/Mn-MOF@BC) and nickel foam (Ni/Mn-MOF@NF) as a visible-light photocatalyst and supercapacitive electrode
Source: Sci Rep. 2023 Nov 7;13:19260. doi: 10.1038/s41598-023-46188-8 (PMC10630428; doi:10.1038/s41598-023-46188-8)
Supplement: Supplementary file 1 — Supplementary Information. [file 41598_2023_46188_MOESM1_ESM.pdf]

Supporting Information:

Ni/Mn metal-organic framework decorated bacterial cellulose (Ni/Mn- MOF@BC) and nickel foam (Ni/Mn-MOF@NF) as a visible-light photocatalyst and supercapacitive electrode

Soheila Ebrahimi-Koodehi[a], Farhad Esmaeili Ghodsi[a*], Jamal Mazloom[a]

[a]*Department of Physics, Faculty of Science, University of Guilan, Namjoo Avenue, P.O. Box 413351914, Rasht, Iran*

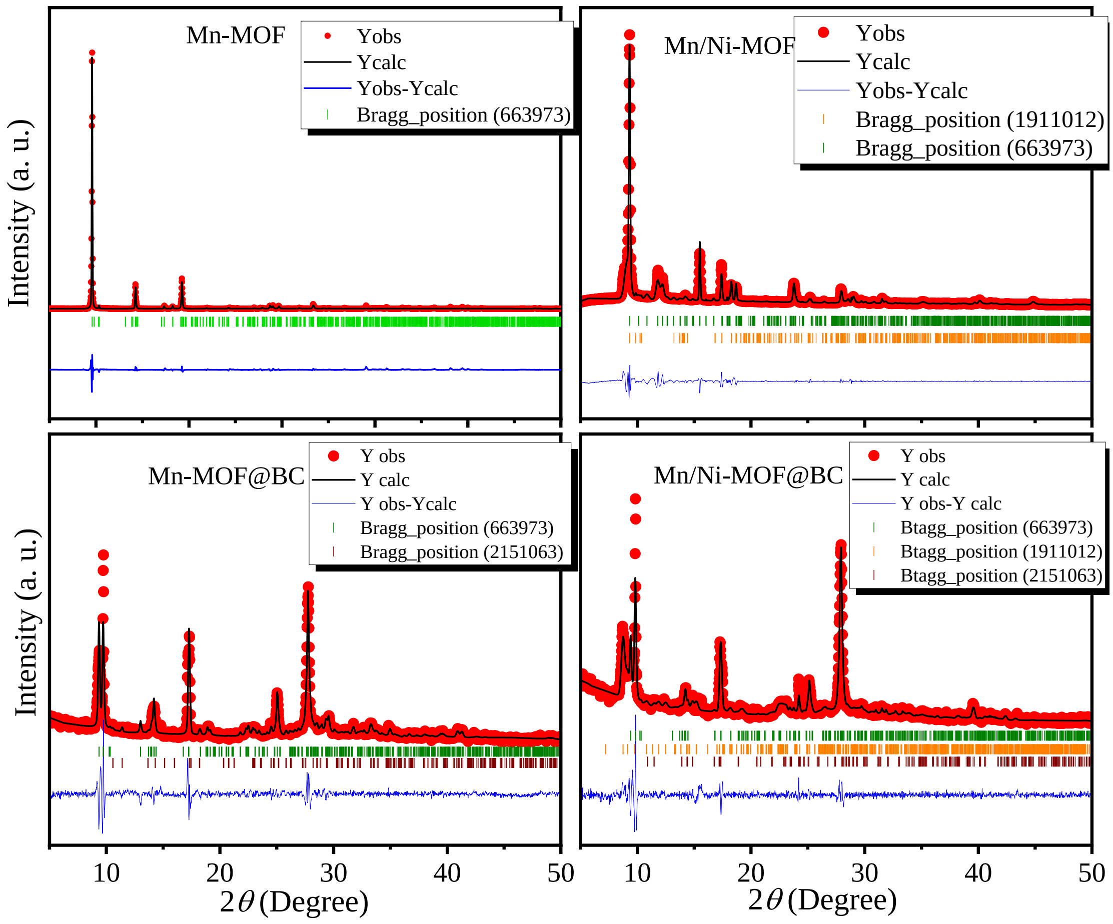

Figure S1: FullProf profile matching of MOFs and MOFs@BC.

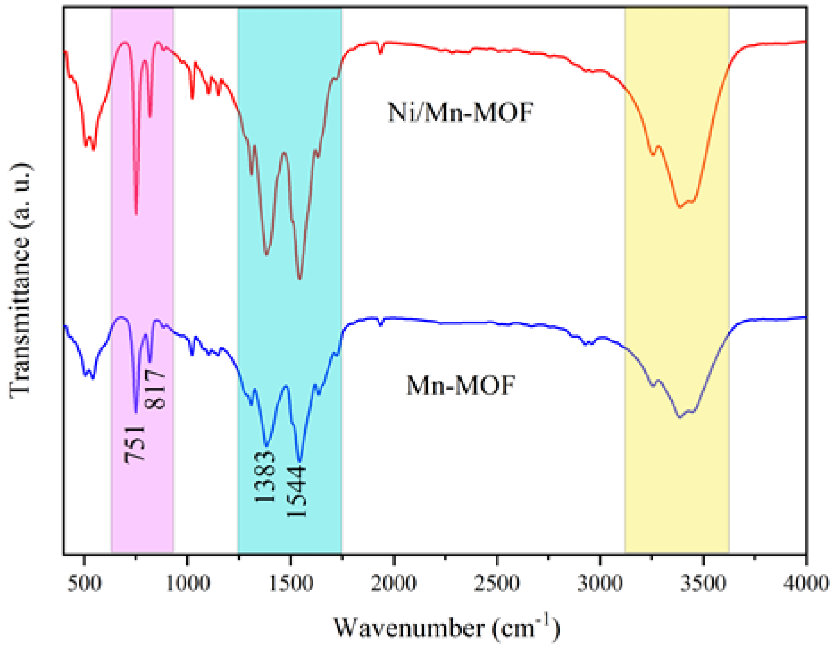

Figure S2: FTIR spectra of Mn-MOF and Ni/Mn-MOF particles.

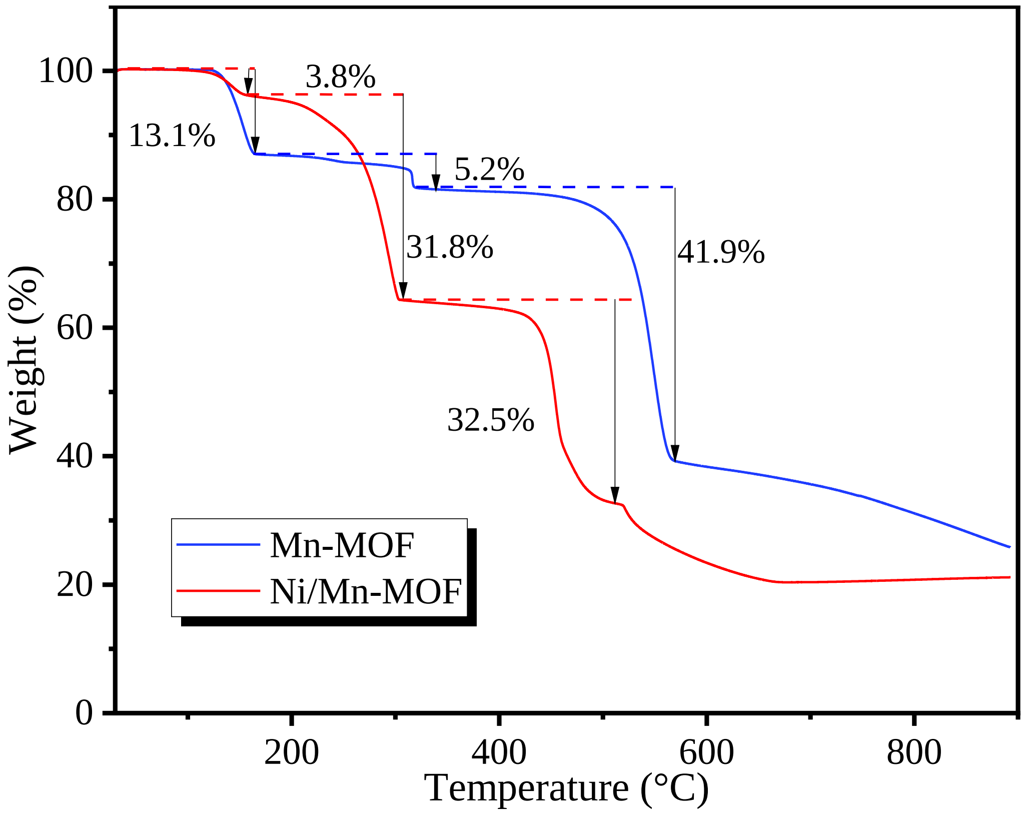

Figure S3: TGA spectra of Mn-MOF and Ni/Mn-MOF particles.

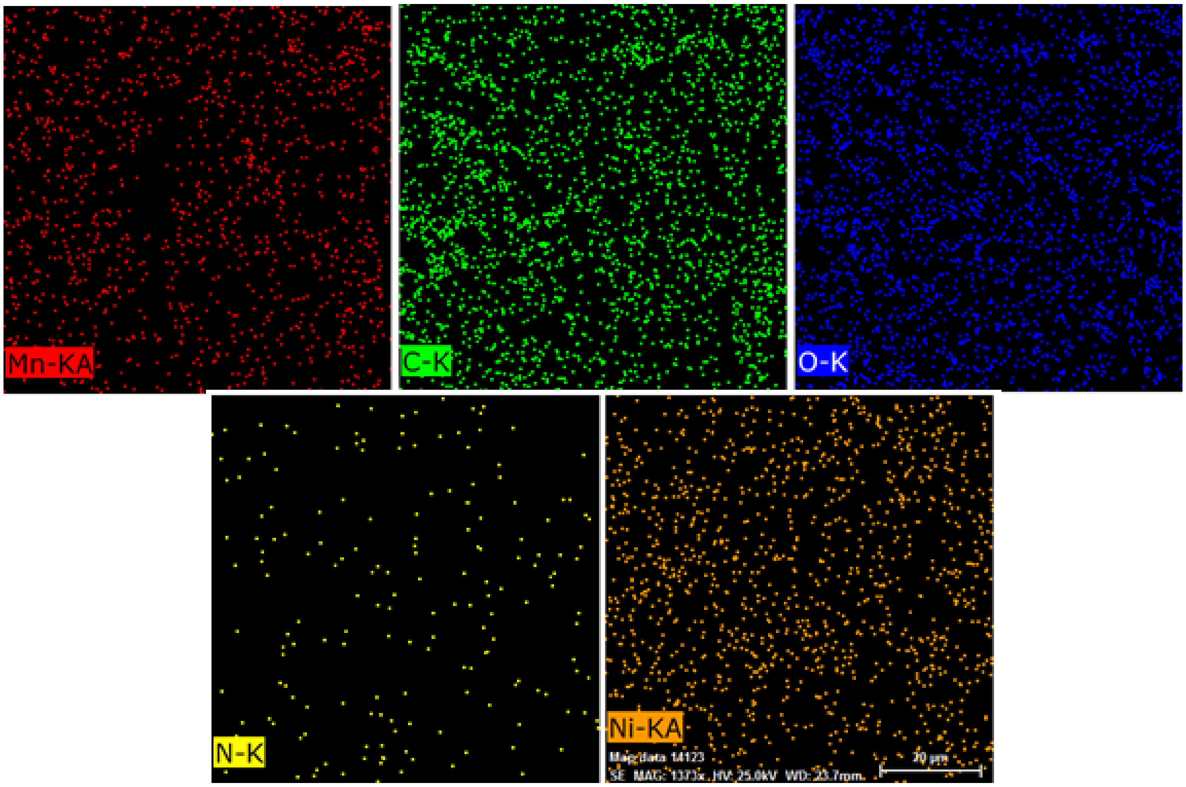

Figure S4. EDS elemental mapping images of Ni/Mn-MOF@BC.

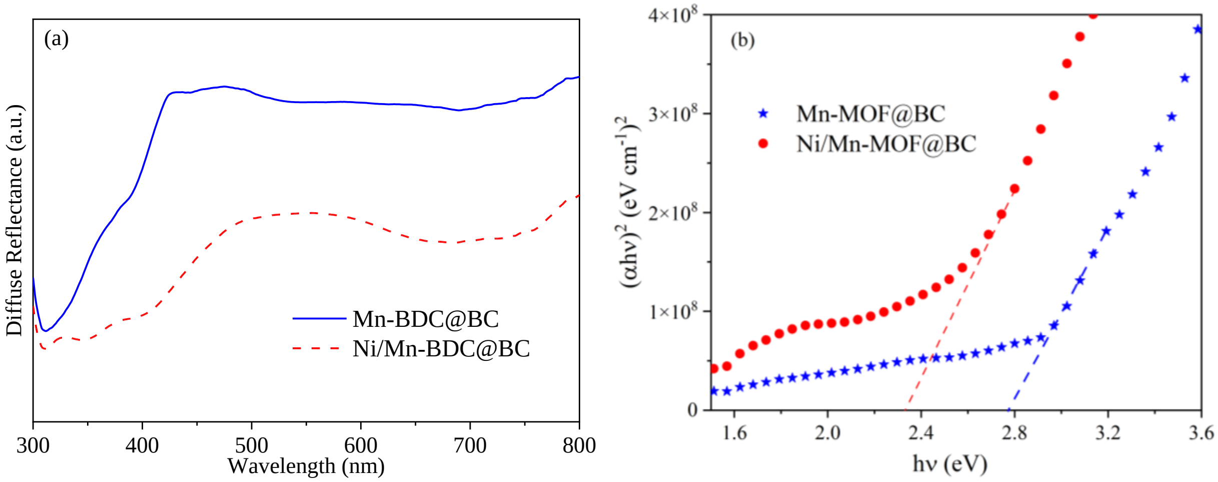

Figure S5. Diffuse reflectance spectra (a), and Tauc's plots (b) for determination of the band gap the samples.

Table S1: comparative study of the obtained specific capacitance of Ni/Mn-MOF@NF and the results of other articles.

Sample	Electrolyte	Specific Capacitance	Current density	Reference
**$Mn_{0.1}$-Ni-MOF/NF**	6M KOH	1178 C g^{-1}	2 mA cm^{-2}	[1]
**Mn/Ni-MOF@MWCNTs**	1M LiOH	793.6 F g^{-1}	1 A g^{-1}	[2]
**MnNiDH**	3M KOH	2498 F g^{-1}	1 A g^{-1}	[3]
**NiCo-MOF@PNTs**	2 M KOH	1109 F g^{-1}	0.5 A g^{-1}	[4]
**Co/Mn MOF**	1 M KOH	1176 F g^{-1}	3 mA cm–2	[5]
**NiCo-LDH/CFC**	2 M KOH	2685 F g^{-1}	1 A g^{-1}	[6]
**$K_{0.5}Mn_2O_4$@Mn-MOF-8**	1M Na_2SO_4	886.9 F g^{-1}	1 A g^{-1}	[7]
**Ni-$ZIF67_{72.75\%}/Mn_{24.25\%}/MWCNT_{3\%}$-LDH**	2 M KOH	2395 F g^{-1}	1 Ag^{-1}	[8]
**Ni/Mn-MOF@NF**	3 M KOH	2769 F g^{-1}	1 A g^{-1}	This work

Reference:

1. Zheng, D., et al. Ultrathin Mn Doped Ni-MOF Nanosheet Array for Highly Capacitive and Stable Asymmetric Supercapacitor. *Chem. Eur. J.* **26**(71), 17149-17155 (2020).

2. Han, Y., et al. Redox-active nanostructure electrode of Mn/Ni bimetal organic frameworks anchoring on multi-walled carbon nanotubes for advanced supercapacitor. *J. Electroanal. Chem.* **882**, 114993 (2021).

3. Liu, H., et al. Spear-shaped Mn/Ni bimetallic hydroxide derived from metal-organic frameworks as electrode materials for aqueous and all-solid-state hybrid supercapacitors. *Colloids Surf.* **601**, 125011 (2020).

4. Liu, H., et al. Spear-shaped Mn/Ni bimetallic hydroxide derived from metal-organic frameworks as electrode materials for aqueous and all-solid-state hybrid supercapacitors. *Colloids Surf.* **601**, 125011 (2020).
5. Seo, Y., Shinde, P. A., Park, S., & Jun, S. C. Self-assembled bimetallic cobalt–manganese metal–organic framework as a highly efficient, robust electrode for asymmetric supercapacitors. *Electrochim. Acta.* **335**, 135327 (2020).
6. Xue, X., Zhong, J., Liu, J., Hou, Z., Wu, X., Li, S., & Yu, M. Hydrolysis of metal-organic framework towards three-dimensional nickel cobalt-layered double hydroxide for high performance supercapacitors. *J. Energy Storage.* **31**, 101649 (2020).
7. Yao, S., Jiao, Y., Sun, S., Wang, L., Li, P., & Chen, G. Vertically Co-oriented Mn-Metal–Organic framework grown on 2D cation-intercalated manganese oxide via a self-sacrificing template process for a high-performance asymmetric supercapacitor. *ACS Sustain. Chem. Eng.* **8**(8), 3191-3199 (2020).
42. Elsonbaty, A., Elshaer, A. M., Harb, M., Soliman, M., Ebrahim, S., & Eltahan, A. Novel ZIF67/Mn/MWCNTs decorated with layer double hydroxide supercapacitor electrodes. *Electrochim. Acta.* **368**, 137577 (2021).

Availability of Data and Materials

The datasets used and/or analysed during the current study available from the corresponding author on reasonable request.
